# Analyzing the Performance of a Miniature 3D Wind Sensor for Mars

**DOI:** 10.3390/s20205912

**Published:** 2020-10-20

**Authors:** Manuel Domínguez-Pumar, Lukasz Kowalski, Vicente Jiménez, Ivette Rodríguez, Manel Soria, Sandra Bermejo, Joan Pons-Nin

**Affiliations:** 1 Micro and Nano Technologies Group, Electronic Engineering Department, Universitat Politècnica de Catalunya, 08034 Barcelona, Spain; lukasz.kowalski@upc.edu (L.K.); vicente.jimenez@upc.edu (V.J.); sandra.bermejo@upc.edu (S.B.); joan.pons@upc.edu (J.P.-N.); 2 Turbulence and Aerodynamics in Mechanical and Aerospace Engineering Research Group, Universitat Politècnica de Catalunya, 08222 Terrassa, Spain; ivette.rodriguez@upc.edu (I.R.); manel.soria@upc.edu (M.S.)

**Keywords:** mars exploration, wind sensors, spherical sensors, thermal anemometry, low pressure atmosphere, heat transfer

## Abstract

This paper analyzes the behavior of a miniature 3D wind sensor designed for Mars atmosphere. The sensor is a spherical structure of 10 mm diameter divided in four sectors. By setting all the sectors to constant temperature, above that of the air, the 3D wind velocity vector can be measured. Two sets of experiments have been performed. First, an experimental campaign made under typical Mars conditions at the Aarhus Wind Tunnel Simulator is presented. The results demonstrate that both wind speed and angle can be efficiently measured, using a simple inverse algorithm. The effect of sudden wind changes is also analyzed and fast response times in the range of 0.7 s are obtained. The second set of experiments is focused on analyzing the performance of the sensor under extreme Martian wind conditions, reaching and going beyond the Dust Devil scale. To this purpose, both high-fidelity numerical simulations of fluid dynamics and heat transfer and experiments with the sensor have been performed. The results of the experiments, made for winds in the Reynolds number 1000–2000 range, which represent 65–130 m/s of wind speed under typical Mars conditions, further confirm the simulation predictions and show that it will be possible to successfully measure wind speed and direction even under these extreme regimes.

## 1. Introduction

The characterization of surface weather in Mars has been and continues to be one of the main science objectives in many Mars missions. As an example, Goal D3 of the Mars 2020 mission (Perseverance rover) is: “*Surface weather measurements to validate global atmospheric models*”. In the 2020 version of the report “*Mars Science Goals, Objectives, and Priorities*”, prepared by the Mars Exploration Program Analysis Group, [1], it is mentioned, in Goal II, Sub-Objective A1, that: “*Obtaining a high-quality dataset from a properly accommodated surface-based weather station (i.e., one in which thermal and mechanical contamination from the spacecraft is minimized beyond what has been done previously) is still of highest priority*”. The main reason for obtaining high quality surface weather measurements is to provide “*vital ground-truth validation for complementary measurements retrieved from orbit and essential data for designing and validating climate and weather model parameterizations*”.

Additionally, it is also pointed out in the same report that obtaining multiple simultaneous datasets collected from multiple locations would contribute to produce major advances in the understanding of the current Martian weather and climate. In summary, a network of surface sensors and/or even sensors placed on aerial platforms, may definitely contribute to the understanding of the Martian atmosphere. Additionally, contamination from the spacecraft, or the supporting structure of the sensor itself, should be carefully minimized. These two objectives are a clear motivation for the miniaturization of wind sensors.

Miniaturization is a key enabling technological factor in space exploration, since it allows cost-reduction [2]. Examples can be found in the use of miniaturized COTS solid-state sensors [3,4,5], or MEMS-NEMS, [6], or even micro-propulsion systems [7,8,9]. Besides the obvious cost reduction, payload miniaturization allows the use of smaller spacecraft, such as penetrators [10,11], or the possibility of placing the sensors in small rovers [12], and landers [13,14]. It may also enable the use of the sensors in aerial platforms [15,16,17,18], or increase the science output in opportunistic missions.

The objective of this paper is to present the latest results of a miniaturized 3D wind sensor [19]. From the Soviet Mars 2 lander to the Mars2020-Perseverance rover, many Mars missions in the past have included a meteorological station or at least a wind sensor. Successful landers/rovers that have provided wind sensor data are Viking, VL-1 and VL-2 [20,21,22], Pathfinder [23,24], Phoenix [25,26], Mars Science Laboratory [27,28,29], and InSight, [30,31,32]. All these sensors measured wind by measuring the convective heat transfer to the airflow, the only exception being the Pathfinder and Phoenix windsocks. In this case it was necessary to point the camera to the telltales in order to observe their deflection and from the images infer wind speed.

Other alternative technologies are being considered in planetary exploration for measuring wind speed. Depending on the specific characteristics of the atmosphere, different options may be offered. Such is the case of Venus, for example, where the very high pressure (93 bar) and high temperatures offer the possibility of using silicon carbide (SiC) strain sensors for wind sensing [33,34]. Additionally, ultrasonic wind sensors are being considered for Mars atmosphere [35]. These sensors generally present high precision and measurement rate, but are difficult to miniaturize (structure weight in the range of 300 g and volume in the range of 10 cm × 10 cm × 10 cm), and their supporting structure may itself interfere with the wind patterns [36], although this shadowing effect can be reduced, in principle, using calibration [35].

The 3D miniaturized wind sensor presented in this paper is an evolution of three wind sensors that have reached Mars. The wind sensors in the REMS (MSL) and MEDA (Mars2020-Perseverance) instruments integrate 2D wind transducers [37], at different points in two booms protruding from the mast of the rover [38]. In the case of TWINS (InSight), the booms are placed on the top deck of the lander. The objective of these transducers is to measure the 2D wind component at different local points on the surface of the boom (3 points/boom in the REMS and TWINS case, and 6 points/boom in the MEDA case). From these tangential wind speeds, 3D wind speed recovery is made.

The core of the REMS, TWINS, and MEDA wind sensors is formed by silicon dice placed on top of 4-leg structures to provide thermal isolation from the boom. The silicon dice contain customized Pt resistors and have been fabricated at the UPC clean room. The thermal isolation and sensing scheme used for the silicon dice at the core of the sensors was first proposed in [39]. Signal routing is accomplished with wire-bonding from the dice to the supporting PCB. The main advantage of this solution is the reduction of the time response [40], since the silicon dice are small (1.5 mm × 1.5 mm × 0.5 mm), have a very small mass, and are placed directly in contact with the airflow.

The main driving forces behind the sensor evolution have been:

- Simplification of the 3D wind component retrieval. The spherical geometry of the sensor greatly simplifies wind speed and angle retrieval, due to its symmetry. The signals generated in the sectors are sinusoidal-like, and it has been possible to design a simple inverse algorithm that will be presented later in the paper.
- Increase robustness, miniaturization and system simplification (wind speed is inferred only from four signals). The sphere containing the active sectors has a 10 mm diameter.

In this miniaturized sensor the actively heated silicon dice have been placed inside a sphere divided in four sectors, with one die attached to each sector [19]. Even though the elements that interact with the wind have more thermal mass than in the case of the previous sensors, it has been possible to optimize the sensor geometry, fabrication process and operation control, in order to provide time responses below 1s [41,42]. Additionally, special care has been taken to avoid parasitic effects, such as heat conduction from the sectors to the supporting stem, which might damage in particular the time response of the sensor.

The main objective of this paper is twofold. First, to present the experimental results in the laminar regime (Reynolds numbers up to 225) obtained with the sensor at the Aarhus Wind Tunnel [43,44], comparing them with Finite Element Method simulations, and applying an inverse algorithm to obtain angles of incidence. And second, to analyze the potential application of this sensor in the dust-devil regime and beyond (Reynolds numbers 1000–2000). High Fidelity simulations of flow around a sphere were described previously in [45]. In the present publication, the heat transfer coefficients calculated from those simulations are compared to experimental data using the sensor prototype. The study in [45] in particular allows the prediction of the specific “signatures” to be expected in the sensor signals. In this paper we present how the experimental measurements match the high-fidelity simulations well. Preliminary versions of some of the results in this paper have been published in [46,47].

The paper is structured as follows. Section 2 describes the structure and basic operation of the sensor. Section 3 presents and discusses the experimental results obtained with the sensor working under typical Martian wind conditions in the Aarhus Wind Tunnel Simulator facility. Then, Section 4 explains the inverse algorithm used to infer the wind direction from the output of the sensor. Section 5 investigates, through both simulations and experiments, the performance of the sensor under extreme Martian wind conditions, with Reynolds numbers reaching and eventually surpassing the Dust Devil scale. Finally, Section 6 draws the main conclusions.

## 2. Sensor Operation

As it has been mentioned above, the 3D wind sensor is a miniaturized sphere of 10 mm diameter composed of four equally shaped sectors. To sense temperature and provide heating power, a customized silicon die, manufactured in-house, which includes a Pt resistor, is attached to each sector. The four sectors are then attached to the core of a supporting structure. To reduce heat transfer between the core and the sectors, the core is kept at the same temperature of the sectors using two additional dice. This strategy is used to avoid a direct heat conduction path between the sectors and the stem.

In order to work as a wind sensor, the constant temperature of all sectors, and cores, must be above that of the surrounding air. To achieve this, the heater dice must provide the power necessary to compensate any thermal conductance variation at each sector caused by external disturbances, such as changes in convection due to the incident wind. This way, the wind speed and direction can be easily inferred from the power injected in the sector resistors.

After the sensor description, in the rest of this section, the principle of operation of the wind sensor is first described and its appropriate operation conditions are derived. Then an approximate model, which allows the prediction of the basic performance of the sensor, is obtained. Finally, the structure and main characteristics of the sensor prototype used in this work are described in detail. The structure and performance of previous versions of the sensor under winds close to Martian conditions have been previously explained in [19], and their dynamics have also been analyzed in [41,42].

### 2.1. Operation Conditions for the Spherical Sensor

Let us analyze here the principle of operation of the spherical sensor and then extract the conditions that, a priori, must be met in order to obtain a proper operation. The Navier–Stokes equations are generally agreed to be valid for *Kn* < 0.01 [48], where the Knudsen number *Kn* is defined as *λ/L,* where *λ* is the mean-free path and *L* a characteristic length scale. In our case, *λ* = 10 μm [49], and thus *Kn* = 0.001. Therefore, a continuum mechanics approach is used here.

**Figure 1:**
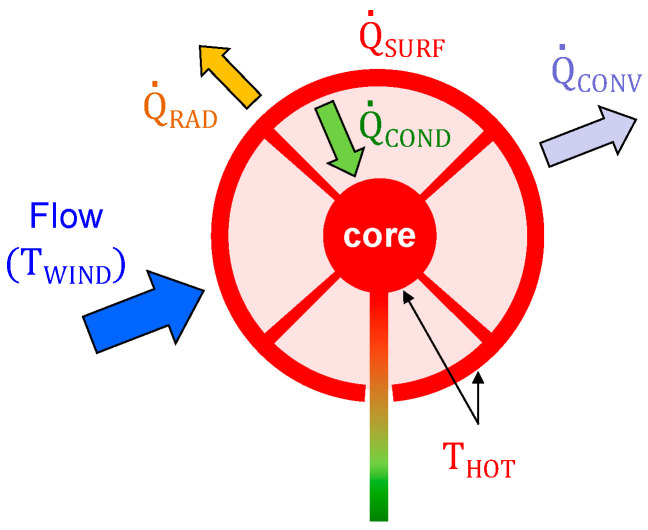
Heat flux components actuating on the surface of the spherical sensor. To minimize the thermal conduction losses between them, the temperatures of both the surface, *T_SURF_*, and of the core of the support structure, *T_CORE_*, are forced to be *T_HOT_*.

Then, let us consider the situation shown in Figure 1, where wind reaches a continuous sphere attached to the core of a small support structure. As mentioned before, the sphere surface is forced to work at a temperature, *T_HOT_*, which is constant and higher than the surrounding air temperature. To maintain this condition, some total heat ${\dot{Q}}_{SURF}$ must be transferred to the sensor surface.

According to the situation described in Figure 1, ${\dot{Q}}_{SURF}$ splits into three flux components:

(1) $${\dot{Q}}_{SURF}={\dot{Q}}_{CONV}+{\dot{Q}}_{RAD}+{\dot{Q}}_{COND}$$

where ${\dot{Q}}_{CONV}$ is heat exchanged between the sphere and the ambient due to the convective heat transfer mechanism, ${\dot{Q}}_{COND}$ is the conduction heat loss to the supporting structure and ${\dot{Q}}_{RAD}$ is the radiation heat loss.

In general, ${\dot{Q}}_{CONV}$ can be not only due to the wind (forced convection) but also due to natural convection. The Richardson number is defined as:

(2) $$Ri=\frac{g\beta \Delta TD}{v^{2}}$$

where g is the Mars surface gravitational acceleration, β the thermal expansion coefficient, ΔT the temperature difference between the sensor and the ambient (ΔT = 40 °C, a typical value in practical applications of the sensor, is used here), D the characteristic length of the sensor (in the case of a sphere, its diameter) and v the wind velocity. For this sensor, and wind speeds above 0.3 m/s, we have *Ri* < 0.1, and therefore natural convection effects can be neglected [50].

The conduction component of Equation (1) is ${\dot{Q}}_{COND}=\left(T_{SURF}-T_{CORE}\right)/R_{th}$, where *T_SURF_* is the temperature of the surface of the sphere and *R_th_* is the thermal conduction resistance between the surface and the core of the structure. By operating the core at constant temperature, these conduction losses are either minimized (*T_SURF_* = *T_CORE_* = *T_HOT_*) or constant (*T_SURF_* > *T_CORE_*). Additionally, the radiation heat component ${\dot{Q}}_{RAD}=\varepsilon \cdot \sigma \cdot \left(T_{SURF}^{4}-T_{AIR}^{4}\right)$ can be strongly reduced by polishing and gold-plating the external surface of the sphere, which then can act as mirror-like with very low emissivity factors (*ε*) down to 0.02–0.03. This also serves to minimize radiation loading on the sensor sphere, since the polished gold-plated surfaces would behave as mirrors, reflecting incident solar radiation.

When these two conditions are met and taking into account Newton’s cooling law, Equation (1) becomes:

(3) $${\dot{Q}}_{SURF}={\dot{Q}}_{CONV}=G_{th}\cdot \left(T_{SURF}-T_{AIR}\right)$$

where *G_th_* is the overall convection heat transfer coefficient, i.e., the convection heat transfer coefficient multiplied by the sphere surface. The conclusion from Equation (3) is that the heat delivered to the surface ${\dot{Q}}_{SURF}$ is transported by convection to the ambient ${\dot{Q}}_{CONV}$ and that *G_th_* is the rate of this phenomenon. As forced convection heat transfer dominates, *G_th_* typically depends on parameters such as wind velocity and pressure. Accordingly, to predict the performance of the sphere working as a wind sensor, it is now necessary to investigate the dependence of *G_th_* on the wind speed and other key parameters of the system.

### 2.2. Modeling the Heat Transfer Response of the Sensor

This section presents an analytical model of the overall convective heat transfer coefficient of the spherical sensor when it is in a fluid under the conditions discussed above. The model is derived from analytical expressions obtained for the average values of three relevant parameters of heat transfer dynamics: Prandtl, Reynolds, and Nusselt numbers.

Thus, for a given fluid or gas, the Prandtl number is defined as the ratio of its momentum diffusivity to its thermal diffusivity and can be calculated as follows:

(4) $$Pr=\frac{C_{P}\cdot \mu }{k}$$

where *μ* is the dynamic viscosity, *k* the thermal conductivity and *C_p_* the specific heat of the gas (in our case CO_2_). All these parameters, and so the *Pr* number, depend only on the gas type and state and therefore they are functions of the temperature.

For its part, the Reynolds number is defined as the ratio of inertial forces to viscous forces within a fluid near a boundary layer. For the case of a sphere within a fluid, the Reynolds number can be calculated as follows:

(5) Re=ρD·Uν=m·PR·T·D·Uν

where *ρ* is the density of the fluid, *ν* is the kinematic viscosity, *D* is the characteristic length of the boundary structure (in our case, the diameter of the sphere, 1 cm) and *U* is the flow or wind speed some distance away from the sphere. Additionally, *P* is the pressure, *T* is the temperature, *m* is the molar mass of the gas (i.e., 44.01 × 10^−3^ kg/mol for CO_2_) and *R* is the gas constant (8.31447 J/(K·mol) for CO_2_). According to Equation (5), in our case the Reynolds number varies with pressure, temperature and wind speed.

Finally, the Nusselt number is defined as the ratio of convective to conductive heat transfer across a boundary. In forced convection cases such as this, the Nusselt number typically depends on Reynolds and Prandtl numbers. Moreover, empirical functions of this kind for a variety of geometries and conditions are available in the literature. Thus, taking the expression for heat transfer from a sphere from [51], valid for low to moderate Reynolds numbers, we have:

(6) $$\mathrm{Nu}=\alpha_{1}Re^{1/3}Pr^{1/3}+\alpha_{2}Re^{2/3}Pr^{1/3}$$

where *α*_1_ = 0.922 and *α*_2_ = 0.1. Note that expression (6) indicates that in our case the average Nusselt number depends only on pressure, temperature and wind speed.

Besides, the Nusselt number can be calculated from its definition as *Nu* = *h·D/k*, where *h* is the convective heat transfer coefficient. From this and being *A_sph_* the area of the sphere, the convection heat transfer coefficient *G_th_* for the whole sphere is obtained as follows,

(7) $$G_{th}=h\cdot A_{sph}=Nu\cdot k\cdot \pi D.$$

**Figure 2:**
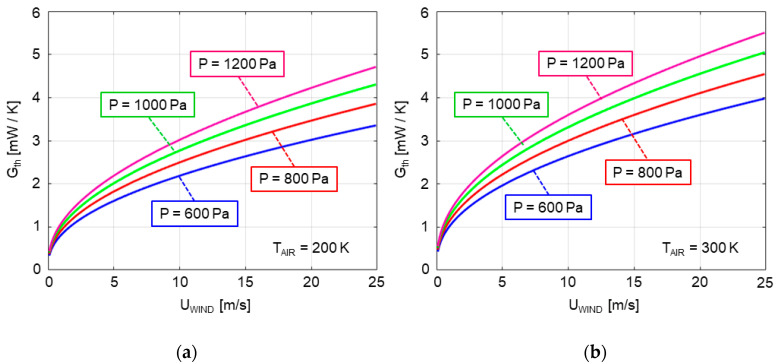
Results of the analytical model developed illustrating the relationship between the overall convective heat transfer coefficient of the sphere and the wind velocity in CO_2_ atmosphere, for two temperatures, 200 K (**a**) and 300 K (**b**), and four different values of pressure.

This model allows obtaining the expected response of the sensor for values of pressure and temperature that are relevant in the Martian environment. The curves in Figure 2 illustrate the monotonic relationship between the overall convection heat transfer coefficient of the sphere and the wind velocity, for two values of temperature (200 and 300 K) and four values of pressure (600, 800, 1000, and 1200 Pa).

### 2.3. Structure, Operation, and Basic Features of the Sensor

**Figure 3:**
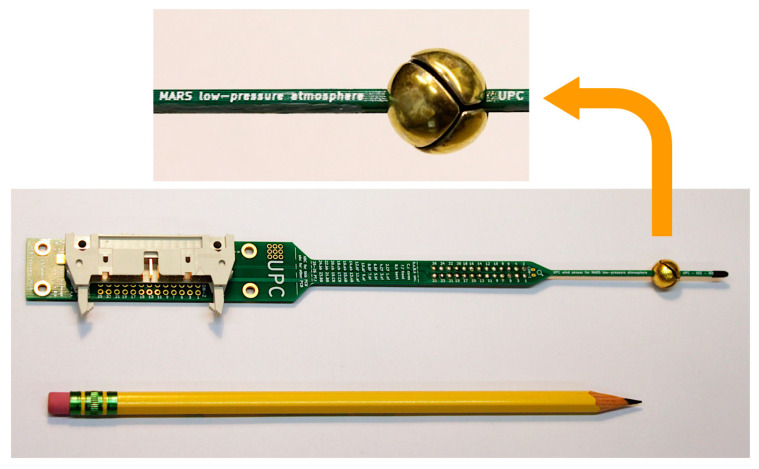
Bottom: Photograph of the sensor with its printed circuit boards (PCB) support structure. Top: Detail of the 10 mm spherical head, showing the shape of the sectors.

The structure of the spherical sensor is shown in the pictures of Figure 3. It is a spherical shell divided into four sectors, obtained using a central projection of a tetrahedron onto the surface of the unit sphere. The sectors are made of silver and, in order to improve the reflectivity of their external surface, they have been polished and a layer of gold has been deposited by sputtering.

To sense temperature and provide heating power, a customized 3 × 3 mm silicon die, which includes a platinum resistor, is attached to the inner side of each sector. The dice are fabricated in the Clean Room facilities of the UPC.

To conform the spherical head, the four sectors are placed on two superimposed printed circuit boards (PCBs), which act as supporting structure, with two sectors on each one. Two additional dice with integrated platinum resistors are placed on the supporting PCBs to control the temperature at the core of the sphere. The PCBs also provide signal routing for the six dice of the sensor. The physical dimensions and mass of the sensors components are summarized in Table 1. Note that the PCB and the connector are optimized for lab measurements, not for space mission constraints.

Two versions of the sensor are used in the experiments reported in this work. In the experiments carried out first, at the Aarhus wind tunnel, the gaps separating the sectors were not covered. On the other hand, while testing the sensor under turbulent conditions, such as those reported in Section 5, it was observed that covering the gaps served to improve the response of the sensor and that the response matched better the predictions of the high-fidelity simulations. In a flight version of the sensor, obviously the gaps would be covered.

**Figure 4:**
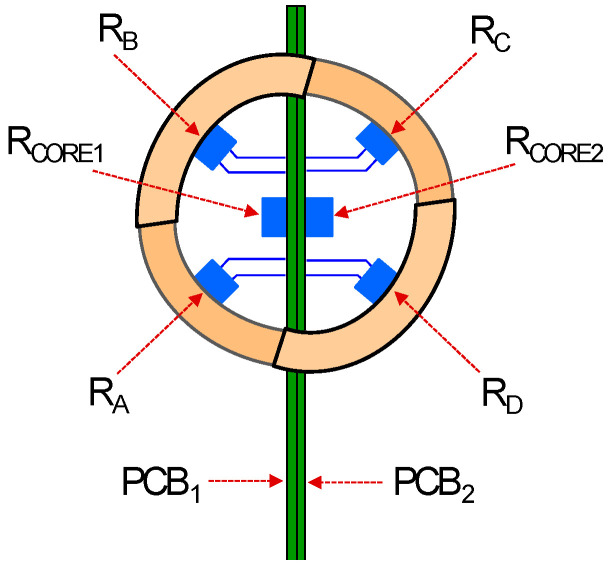
Detail of the two PCB, four-sector and two-core structure of the spherical head.

The overall organization of sectors, PCBs and Pt resistors in the sensor head is described in Figure 4. There, *R_A_*, *R_B_*, R_C_, and *R_D_* are the four sector resistors and *R_CORE1_* and *R_CORE2_* the core resistors on each PCB. These six resistors are forced to operate at the same constant temperature, therefore enforcing *T_CORE_* = *T_SURF_* = *T_HOT_*. This way, heat transfer between all sensor head components is minimized.

The output signals of the sensor are the heating powers *P_i_* injected in the four resistors. Assuming that these powers compensate convection losses to the ambient to keep the temperature of the surface constant, 3D wind speed recovery is made taking into account only these powers and the ambient temperature, *T_AIR_*, and pressure. For instance, the wind speed is inferred from the overall convective heat transfer coefficient of the sphere, *G_th_*, which is calculated as:

(8) $$G_{th}=\frac{P_{A}+P_{B}+P_{C}+P_{D}}{T_{SURF}-T_{AIR}}.$$

Additionally, the angle information, as it will be seen later, can be inferred from linear combinations of the normalized sector powers:

(9) $${\bar{P}}_{i}=\frac{P_{i}}{{\left||P_{A}-P_{av},P_{B}-P_{av},\;P_{C}-P_{av},\;P_{D}-P_{av}|\right|}_{2}}\;i=A,B,C,D$$

where ${\left||\cdot |\right|}_{2}$ is the standard Euclidean norm and $P_{av}=\left(P_{A}+P_{B}+P_{C}+P_{D}\right)/4$. In its intended standalone operation, the sensor will be interfaced and controlled by an application-specific integrated circuit (ASIC), which is currently under development. However, in the laboratory tests reported in this work, the interface has been implemented with a specific LabView program running on a computer equipped with two National Instruments data acquisition cards. This program includes a smart control loop for each sector and core resistor, whose objective is to supply the current (that is, the power) necessary to achieve and maintain its temperature (that is, its resistance) to the desired, or target, value, *T_HOT_*.

More concretely, the control loop periodically samples the value of the resistance and applies a pulse width modulated (PWM) current signal (with two values, *I_HIGH_* and *I_LOW_*) to reach and maintain the target value. The average power supplied is obtained from the duty cycle of the PWM signals applied, as the resistance value is set by the control loop, and therefore has a known value. Additionally, the program continuously monitors other relevant parameters of the environment, such as the pressure and the ambient temperature.

The control of the operation temperature of the sensor (*T_HOT_*) to be implemented will follow the general scheme implemented for the MEDA wind sensor. The main idea behind this control is to adapt and change *T_HOT_* as a function of time in order to keep the sector powers in a desired operational range, in particular avoiding the saturation limits (zero or maximum available power). To this effect, the instantaneous powers are to be continuously monitored, and when certain low and high thresholds are trespassed in a sector, then the operation temperature is increased or decreased accordingly. Programming and optimizing this control strategy will be the object of future work.

Let us note that sensor components inevitably have a certain tolerance. For this reason, it is necessary to carry out a calibration process. This calibration consists of obtaining the exact values of all resistances of the spherical head of the sensor (sectors and cores) at a reference temperature. Calibration identifies each individual sensor and should always be considered before operation.

The authors would also like to point out that a possible strategy for avoiding effects such as radiation loading or the dependence of the measurement on the air temperature is to simultaneously use two spheres, placed relatively close but without generating shadowing, with their sectors placed in the same relative position and working at different target temperatures. In this case, environmental conditions such as the air temperature (*T_AIR_*), the wind, the amount of deposited dust and the solar radiation received would be the same in both spheres. According to this, the powers *P*_1_ and *P*_2_ applied to two sectors placed in the same relative position of each sphere would be,

(10) $$P_{1}=G_{th}\cdot \left(T_{HOT1}-T_{AIR}\right)-P_{SUN}\;P_{2}=G_{th}\cdot \left(T_{HOT2}-T_{AIR}\right)-P_{SUN}$$

where *T_HOT1_* and *T_HOT2_* are the target temperatures of each sphere and *P_SUN_* is the radiation loading. Subtracting these two equations, it yields *P*_1_ − *P*_2_ = *G_th_* (*T_HOT1_* − *T_HOT2_*). Then,

(11) $$G_{th}=\frac{P_{1}-P_{2}}{T_{HOT1}-T_{HOT2}}.$$

The conclusion from Equation (11) is that the sensor spheres would be working in a “differential mode” that allows a first order cancellation of both *T_AIR_* and *P_SUN_*. This new strategy is the subject of current research by the authors.

The total power consumption of the six resistors that compose the spherical head of the sensor, which generally depends on the overheat chosen $\Delta T=T_{HOT}-T_{AIR}$, is estimated to be below 300 mW in most practical applications. For instance, in the experiments under laminar regime reported in Section 3, ΔT = 49 °C is used and the total power consumption is 222 mW. In the experiments under turbulent regimes reported in Section 5, the total power is 228 mW for the case with Reynolds number Re = 1000 (ΔT = 13 °C is used) and 268 mW for Re = 2000 (where ΔT = 10.8 °C). On the other hand, the power consumption of the ASIC is estimated to add less than 200 mW to the total power requirements. In case of using two spheres, the power consumption would increase by another 300 mW, to account for the consumption of the second sphere.

The humidity content in the ambient while performing the experiments was negligible. In the first set of experiments, with the Aarhus wind tunnel, first the chamber is placed in a high vacuum and, in a second step, filled with carbon dioxide. In the second set, performed at UPC, dry air was used.

Finally, as it has been stated in the introduction, this sensor is the evolution of the REMS, TWINS and MEDA wind sensors. In all these sensors, 2D tangential wind transducers are placed at different points of the boom (3 points for REMS and TWINS, 6 points for MEDA). The airflow around the boom generates tangential winds patterns on the transducers, from which the wind is inferred. REMS and TWINS employ 30 silicon dice and MEDA 60. The sensor in this work uses 6 silicon dice, and in case of using two spheres, this number would be 12. In this regard, it is expected a reduction of the necessary data volume.

## 3. Experiment Set 1: Sensor Performance under Typical Martian Conditions

As commented above, this section presents and discusses the experimental results obtained with the spherical wind sensor in the Aarhus Wind Tunnel Simulator II (AWTSII). The main objective of this experimental campaign has been to measure wind velocity for a wide range of yaw and pitch angles, within an environment that reproduces typical Mars conditions. The observation of the time response of the sensor against sudden wind changes and the comparison of its response to empirical models found in the literature are also objectives of these experiments.

### 3.1. Experimental Setup

The AWTSII wind tunnel is one of the few Mars environment simulators available in Europe for instrument research and development, calibration and test for Aerospace applications. This facility is located in the Mars Simulation Laboratory at Aarhus University, Denmark. It is a recirculating wind tunnel inside a cylindrical vacuum chamber of 2.5 m diameter and 8 m long, that enables reproduction of ambient conditions in the surface of Mars: low-pressure CO_2_ atmosphere, wind speeds in the range from 0 to 26 m/s and variable dust content [44,52]. It also features a liquid Nitrogen system, which provides cooling down to the low temperatures typical on Mars surface.

**Figure 5:**
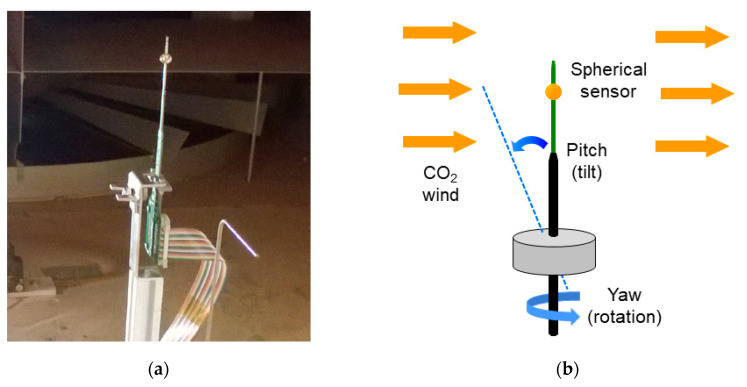
(**a**) Sensor support and orientation structure used in the AWTSII experiments; (**b**) Reproducing 3D wind direction through sensor orientation.

As shown in Figure 5, in our experiments the spherical sensor was mounted on a mobile base, which allows changing the orientation of the sensor relative to the wind. Each pair of pitch and yaw angle determines an angle of incidence on the sphere. A detailed description of the relation between the angles of incidence of the wind on the sphere, and the set of yaw and pitch points swept will be presented later in Section 4.

The environmental conditions targeted in our experiments include CO_2_ pressure around 7–10 mbar, temperatures from −25 to 0 °C and wind velocities spanning the range 0–16 m/s. These conditions correspond to laminar regimes with Reynolds numbers below 225. The angle span is 0–360° full yaw rotation, and pitch angles from −45° to +45°.

### 3.2. Results and Discussion

The objective of the first experiment is to verify that the spherical sensor allows measuring wind speed in Mars-like conditions. In particular, to test how the wind speed can be inferred from the total power delivered to the four sectors, necessary to keep them at the constant target temperature *T_HOT_*. Besides, in order to test the invariability of the results, an experiment at two different target temperatures *T_HOT_* has been performed. The ambient conditions of the experiment are: CO_2_ atmosphere and pressure 7.6 mbar. The sensor was placed vertically, with pitch = yaw = 0°.

**Figure 6:**
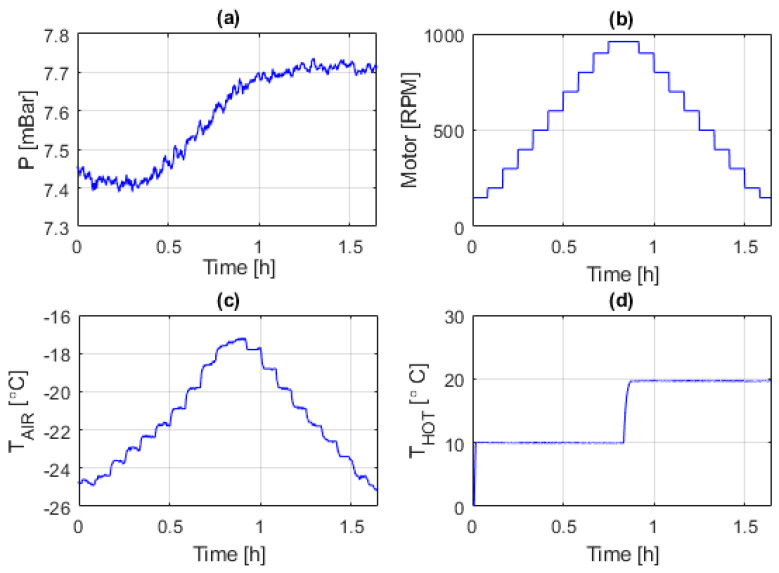
Time evolution, during the first experiment, of: (**a**) The pressure in the wind tunnel; (**b**) rotation speed of the fan in the wind tunnel; (**c**) temperature of the air; (**d**) temperature in the sphere sectors. The wind velocities tested were: 1.5, 2, 3.6, 5.6, 7, 9, 10, 12, 14, 16 m/s.

**Figure 7:**
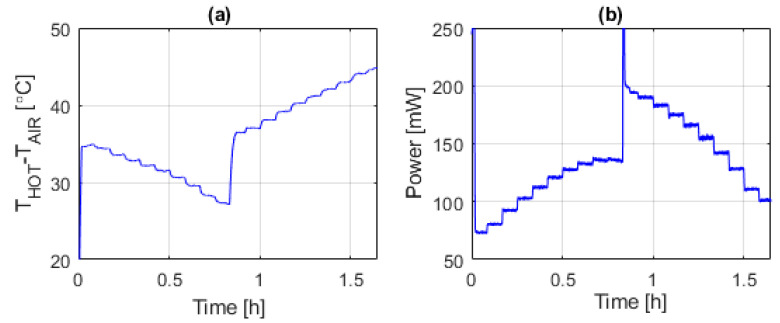
Time evolution, during the first experiment, of: (**a**) The sensor overheat, (**b**) total power injected in sectors.

The time evolution of the pressure and temperature in the chamber were continuously monitored and recorded during the experiment. This is shown in Figure 6a,c, while Figure 6b presents the rotation speed of the fan and Figure 6d shows the evolution of the temperature measured in the sectors of the sphere. Figure 7a shows the temperature difference between the sectors and that of the air, and Figure 7b shows the total power being delivered to the sectors during the experiment.

**Figure 8:**
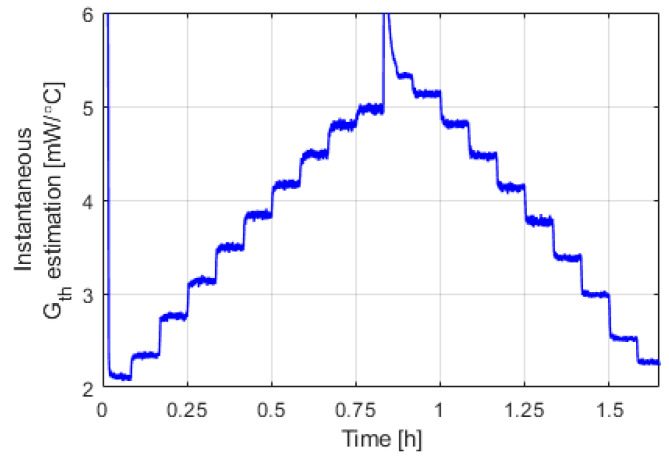
Time evolution of the estimated instantaneous total convective heat transfer coefficient of the sphere, *G_th_*, during the experiment of Figure 6 and Figure 7.

Figure 8 shows the time evolution of the estimated instantaneous total convective heat transfer coefficient of the sphere, *G_th_*, during the experiment.

**Figure 9:**
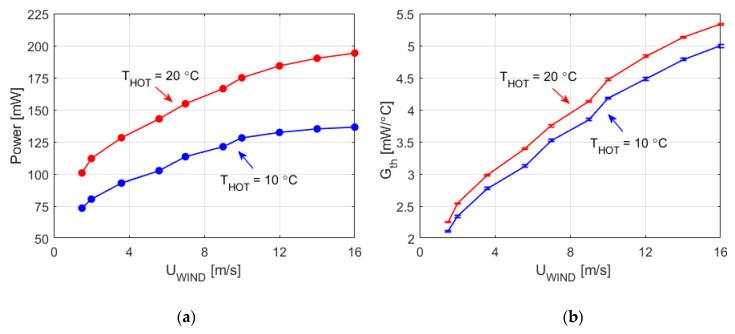
(**a**) Average values of the power injected in all sectors in the experiment of Figure 6 and Figure 7; (**b**) average total convection heat transfer coefficient of the sphere, *G_th_*, during the same experiment, including the 1σ error bars associated to the noise of the samples.

Figure 9a shows the total heating power in the four sectors of the sphere as a function of the wind velocity measured for *T_HOT_* = 10 °C and 20 °C. As expected, a monotonic relationship between the heating power and the wind velocity is observed for each operation temperature. Additionally, Figure 9b shows the overall convective heat transfer coefficient of the sphere, *G_th_*, as a function of the wind velocity, where *G_th_* is calculated taking into account the total heating power delivered to the sectors, the ambient temperature and the pressure variation during the experiment.

**Figure 10:**
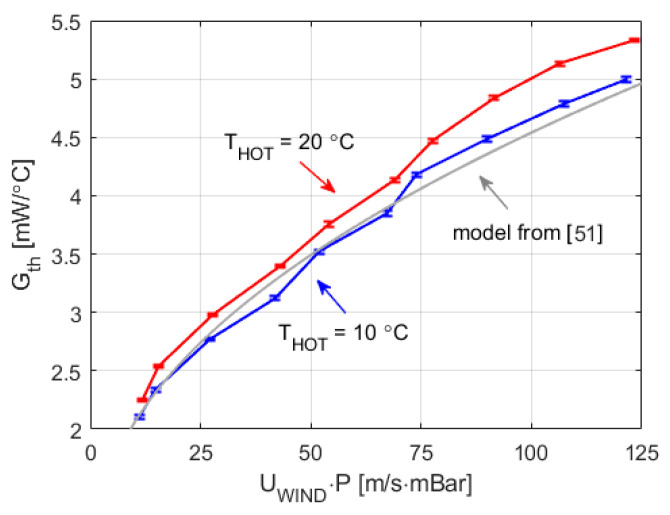
Overall convective heat transfer coefficient, as a function of the product of the wind velocity and pressure, for the experiment of Figure 6 and Figure 7, including the 1σ error bars associated with the noise of the samples. The solid-grey line is the result of evaluating the correlation (6) [51], at T = 300 K for the values of our experiment.

Finally, Figure 10 presents the estimated values of *G_th_* during the experiment, as a function of the product wind velocity and pressure. Figure 10 also includes a comparison with *G_th_* values obtained using the model presented in [51] (solid-grey line). A good agreement exists between the values for *G_th_* obtained from our experiments and those taken from the literature. We can also conclude that, under the Mars-like conditions tested, the relation between *G_th_* and the wind speed is monotonic and has small dependence on the temperature, *T_HOT_*, at which the sensor operates. These results confirm that sensor geometry and constant temperature operation enable easy wind speed recovery from the power delivered.

The second set of experiments performed aims to investigate the ability of the spherical sensor to detect wind from various 3D directions. To this end, the power delivered to each sector and each core was measured while varying the yaw angle from 0 to 360° in 22.5° steps. Seven full rotation experiments of this kind, for pitch angles varying between −45° and 45° in 15° steps, have been performed. The ambient conditions are: CO_2_ atmosphere with pressure 10.3 mbar, temperature −1 °C and constant wind speed of 5.6 m/s. The Reynolds number corresponding to these conditions is approximately 82.15. The operation temperature of the sphere is *T_HOT_* = 48 °C.

**Figure 11:**
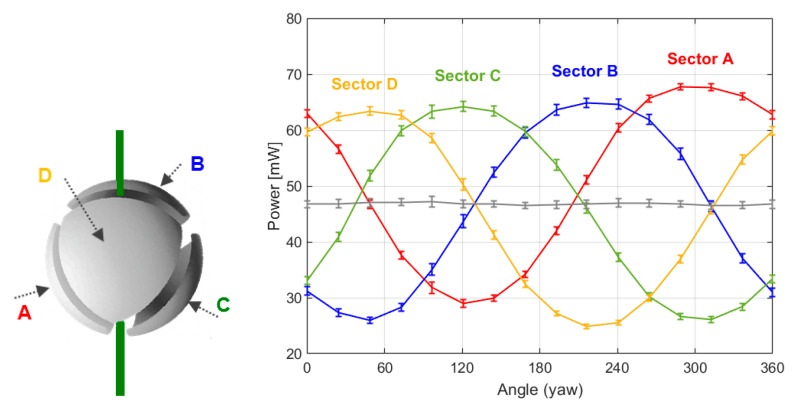
(**Left**) Diagram showing the different sectors names in the sphere. (**Right**) Power delivered to all Pt resistors of the sensor (sectors A, B, C, D) in an experiment in which 0–360° yaw angles were swept for pitch angle = 0, at constant wind speed of 5.6 m/s. Steps of 22.5° in Carbon Dioxide atmosphere. P = 10.3 mbar, U = 5.6 m/s, T = −1 °C, Thot = 48 °C. The grey line is the average power of the sectors. The 1σ error bars of the measurements are also shown.

Figure 11 shows the results obtained for a pitch equal to zero and a complete yaw rotation. There, it is clearly observed that the evolution of the power delivered to each sector with the yaw angle (i.e., with the horizontal direction of the wind) has an almost sinusoidal shape, with constant amplitude. These results clearly indicate that the direction of the wind in both horizontal and vertical planes can be easily inferred from the heating powers injected on each sector.

The average power per sector, (*P_A_* + *P_B_* + *P_C_ + P_D_*)/4, is almost constant (as should be expected) at 47 mW throughout the experiment. According to the theoretical model expectations shown in Figure 2, and also to the experimental results of Figure 6, Figure 7, Figure 8, Figure 9 and Figure 10, this value depends only on the wind speed and pressure, which were kept constant during the experiment. Additionally, the power delivered to the two core resistors is also almost constant at a value of 17 mW each. These core resistors are used to isolate the dice at the sectors from the supporting structure. Variations of the heat flow along the supporting structure are therefore compensated by these core dice.

**Figure 12:**
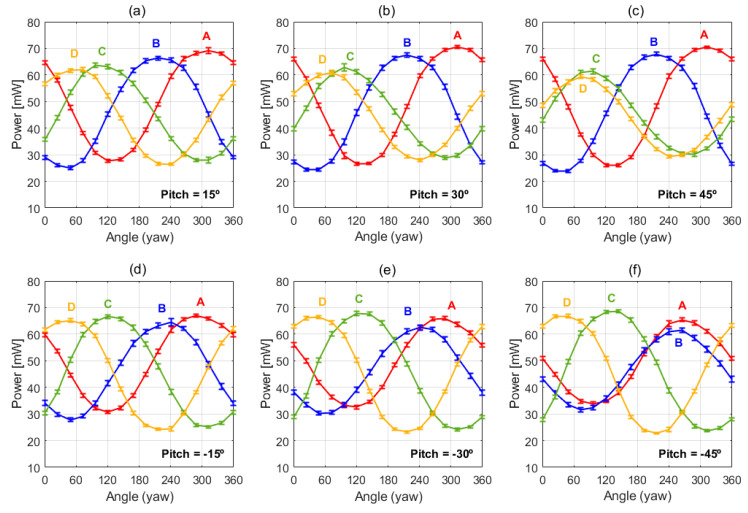
Power delivered to the four sector resistors of the spherical sensor for six non-zero pitch angles and complete yaw rotations, in the same conditions as the experiment reported in Figure 11. Pich angles are: (**a**) 15°, (**b**) 30°, (**c**) 45° and (**d**) −15°, (**e**) −30°, (**f**) −45°. The 1σ error bars of the measurements are also shown. The sectors (A, B, C and D) follow the same disposition as the one shown in Figure 11 (Left).

Figure 12 summarizes the experimental results obtained for the non-zero pitch angles. For a given pitch value, the power delivered to the sectors versus the yaw angle has again a sinusoidal-like shape, therefore enabling the inference of the wind direction in both the horizontal and vertical planes.

It is also observed in Figure 12 that pitch variation does not affect the sinusoidal behavior but produces noticeable amplitude variation. For instance, it can be seen that two of the sectors tend to slightly increase their amplitudes with increasing pitch angles, while the other two tend to reduce it. Moreover, the sinusoids of the two sectors reducing their amplitude tend to approach, and even to cross, for increasing values of the pitch angle. On the other hand, the average power delivered to each sector, linked to the speed of the incident wind, continues to remain constant at around 47 mW.

In order to check the consistency of the experimental results obtained, simulations of the sensor have been carried out with finite element software, reproducing the Mars-like conditions of the experiment. The geometry that has been simulated is that of a complete sphere (imposing therefore uniform temperature boundary conditions on its surface) immersed in the flow. In order to obtain the powers in the sectors, integrals of the heat flow are performed only in the areas of each sector (avoiding the gaps between the sectors).

**Figure 13:**
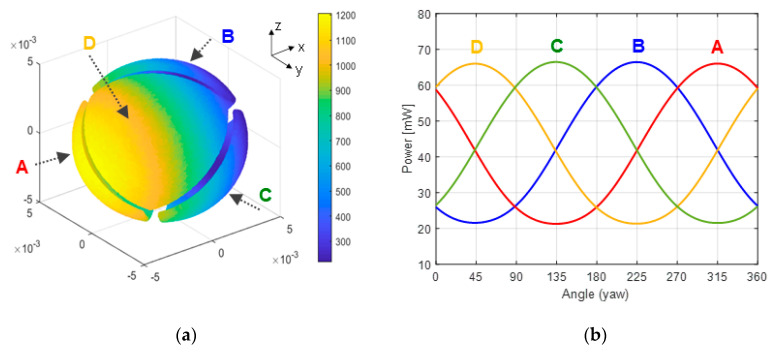
(**a**): Sensor geometry used in the simulations and local heat flux (W/m^2^) for ΔT = 49 °C obtained on the surface when the incident wind is parallel to the *x*-axis. (**b**) Power delivered to each sector as a function of the yaw angle (rotation around the *z*-axis). The sectors (A, B, C and D) follow the same disposition as the one shown in (a) or Figure 11 (Left).

Figure 13a describes the geometry of the spherical sectors considered in the simulations. It also shows the distribution on the surface of the sensor of the local heat flux obtained for an incident wind parallel to the *x*-axis.

To obtain the average convective heat transfer coefficient *G_th_* on a sector as a function of the yaw angle (and therefore the amount of power that must be supplied to the resistor of the sector to keep it at the target temperature *T_HOT_*), separate integration of the heat flux on each sector has been performed. As a result of this strategy, Figure 13b shows the power injected on each sector for a complete yaw rotation and a pitch angle equal to zero. Note that both the shapes and the values of these simulation results are very close to their experimental counterparts reported in Figure 11.

**Figure 14:**
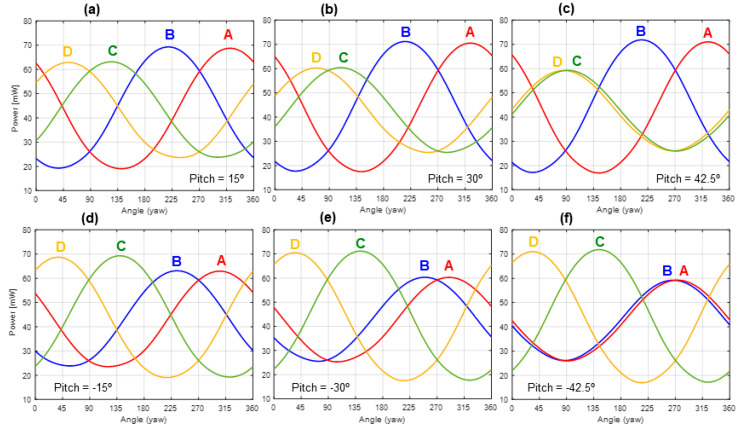
Simulation results showing the power delivered to the four sector resistors of the sensor for complete yaw rotations and six different pitch angles. Pich angles are: (**a**) 15°, (**b**) 30°, (**c**) 42.5° and (**d**) −15°, (**e**) −30°, (**f**) −42.5°. The sectors (A, B, C and D) follow the same disposition as the one shown in Figure 13a or Figure 11 (Left).

Additionally, Figure 14 shows the evolution of the power delivered to each sector with the yaw angle, for six different pitch angles. The same type of behavior as in Figure 12 is clearly observed: Two of the sectors increase their amplitude while the other two decrease it, when the pitch angle increases its absolute value. It is also observed that the two sectors reducing their amplitude “get closer” and even intersect for pitch angles of ±45°.

The geometry of the device allows explaining this behavior. For example, the amplitude increase in sectors A and B for increasing pitch angles up to 45° is due to the larger area exposed to the wind in these two sectors. Symmetrically, sectors C and D are in the “shadow of the wind”, thereby reducing their exposed areas and their power amplitudes for increasing pitch angles. In the case of Figure 14c,f the plots have been obtained for a slightly reduced pitch angle (42.5° and −42.5°, respectively). Otherwise, the curves for sectors C and D would overlap in the +45° case, as well as the curves A and B for −45°.

Based on these results, we can affirm that the simulations allow corroborating and explaining the experimental results obtained. The slight differences observed can be due to some approaches used in the simulations (for example, the effect of the PCB supporting the structure is not taken into account) and small misalignments in sensor orientation.

**Figure 15:**
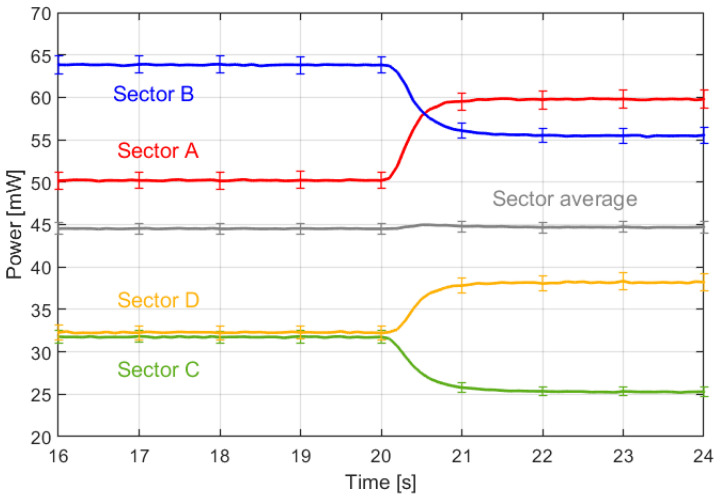
Response time, in the range 0.5–1 s, of the power delivered to the four sensor sectors after a sudden change of the yaw angle (e.g., the wind direction) at *t* = 20 s. The grey curve is the average power delivered to the four sectors. In order to reduce noise, the curves have been averaged from the results of 250 repeated transients. The 1σ error bars of the measurements are also shown. The measurement was made at the UPC wind tunnel, using the sensor with the grooves covered, as in the experiments of Section 5. The sectors (A, B, C and D) follow the same disposition as the one shown in Figure 13a or Figure 11 (Left).

Finally, a last experiment focused on the dynamics of the sensor when working in Mars-like conditions. Previous studies of dynamics characterization and optimization for different versions of the sensor are available in [41,42]. These works indicate that closed loop control enforcing constant temperature operation implies a strong reduction of the response time of the sensor. The transient curves shown in Figure 15 illustrate the average response of the four sensor sectors when a sudden 50° yaw angle change is made at *t* = 20 s (in 0.1 s). The response time (taken as the time necessary to reach the 90% of the steady-state value) is nearly the same, around 0.7 s, for all sectors. Let us also note that the average power remains constant during the experiment, thus indicating that the wind speed did not change.

Summarizing, the experimental results obtained in typical Mars-like ambient conditions reveal that, when working at constant temperature under these laminar regimes, the spherical sensor provides fast, easy and reliable recovery of both speed and solid angle of the incident wind. Additionally, the obtained responses agree with finite element simulations of the sensor and also with empirical models taken from the literature.

## 4. Wind Recovery: Inverse Algorithm for Laminar Regimes

**Figure 16:**
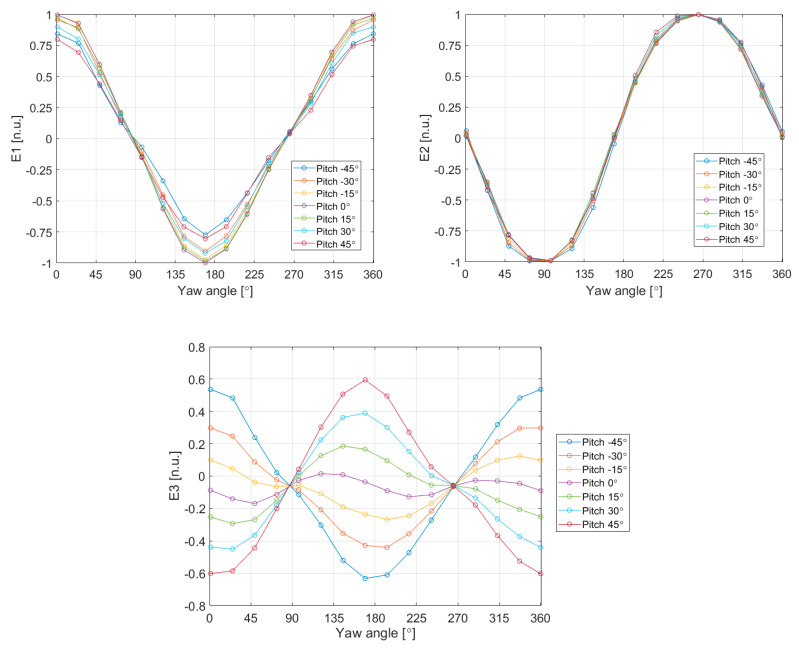
Estimators E1, E2, and E3 for the experiment reported in Figure 11 and Figure 12, as a function of the yaw angle for seven different values of the pitch angle.

Let us now focus on the retrieval of the wind direction from the power data provided by the sensor. An inverse algorithm for the wind angles, based on the geometrical properties of the sphere, has been designed for this purpose. To estimate the angles of incidence of the wind on the sphere, the three following estimators are used:

(12) $$\begin{array}{l} E_{1}=\frac{1}{2}\frac{P_{A}-P_{B}-P_{C}+P_{D}}{\sqrt{{\left(P_{A}-P_{av}\right)}^{2}+{\left(P_{B}-P_{av}\right)}^{2}+{\left(P_{C}-P_{av}\right)}^{2}+{\left(P_{D}-P_{av}\right)}^{2}}}\\ E_{2}=\frac{1}{2}\frac{P_{A}+P_{B}-P_{C}-P_{D}}{\sqrt{{\left(P_{A}-P_{av}\right)}^{2}+{\left(P_{B}-P_{av}\right)}^{2}+{\left(P_{C}-P_{av}\right)}^{2}+{\left(P_{D}-P_{av}\right)}^{2}}}\\ E_{3}=\frac{1}{2}\frac{-P_{A}+P_{B}-P_{C}+P_{D}}{\sqrt{{\left(P_{A}-P_{av}\right)}^{2}+{\left(P_{B}-P_{av}\right)}^{2}+{\left(P_{C}-P_{av}\right)}^{2}+{\left(P_{D}-P_{av}\right)}^{2}}} \end{array}$$

where $P_{av}=\left(P_{A}+P_{B}+P_{C}+P_{D}\right)/4$. Figure 16 shows the values of the estimators E1–E3 for the Aarhus experiment reported in Figure 11 and Figure 12, where complete yaw angle rotations for several different pitch angles were performed.

From the above estimators E1–E3, the polar and azimuthal angles in the spherical coordinates of the local coordinate system of the sphere are, respectively:

(13) $$\begin{array}{c} \bar{\theta_{s}}=cos^{-1}\left(-E_{3}\right)\\ \bar{\phi_{s}}=Arg\left\{E_{1}-i\cdot E_{2}\right\} \end{array}$$

In order to evaluate the accuracy of the measurements, it is necessary to compare the measured values with the ideal local angle retrievals from the pitch and yaw sweeps. This means that the set of different yaw and pitch angles must be converted to angles of incidence on the sphere. This is done using:

(14) $$\begin{array}{c} \theta_{s}\;\left(\mathrm{yaw},\mathrm{pitch}\right)=cos^{-1}\left[\cos\left(\mathrm{yaw}\right)\cdot \sin\left(\mathrm{pitch}\right)\right]\\ \phi_{s\;}\left(\mathrm{yaw},\mathrm{pitch}\right)=Arg\left\{\cos\left(\mathrm{yaw}\right)\cdot \cos\left(\mathrm{pitch}\right)+i\cdot \sin\left(\mathrm{yaw}\right)\right\}. \end{array}$$

**Figure 17:**
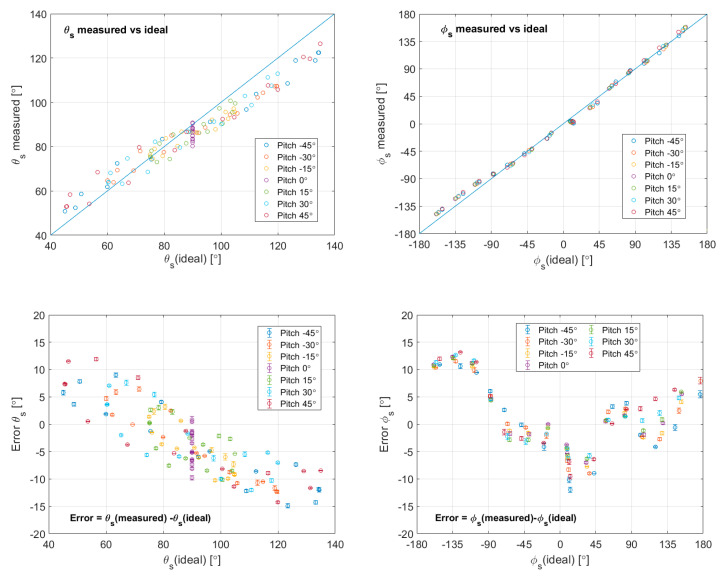
**Top**: estimated vs. ideal values of the angles of incidence on the sphere for the Aarhus experiment reported in Figure 11 and Figure 12. **Bottom**: errors in the above estimations. Each point is presented with the 1σ noise bars associated with the measurement.

Now, it is possible to compare the angles of incidence on the sphere obtained using the estimators E1–E3, with the angles of incidence on the sphere determined by the yaw and pitch points of the experiment. Figure 17, shows the inferred angles of incidence of the wind, as a function of their ideal values, for all the pitch and yaw sweeps in the experiment of Figure 11 and Figure 12. This figure also shows the error in these angle estimations and the associated error bars due to electronic noise.

Looking at the errors in the estimation, it can be stated that the angle accuracy in this experiment, using this simple inverse algorithm, is 13°. The resolution associated with the 1σ error bars is below 2°.

**Figure 18:**
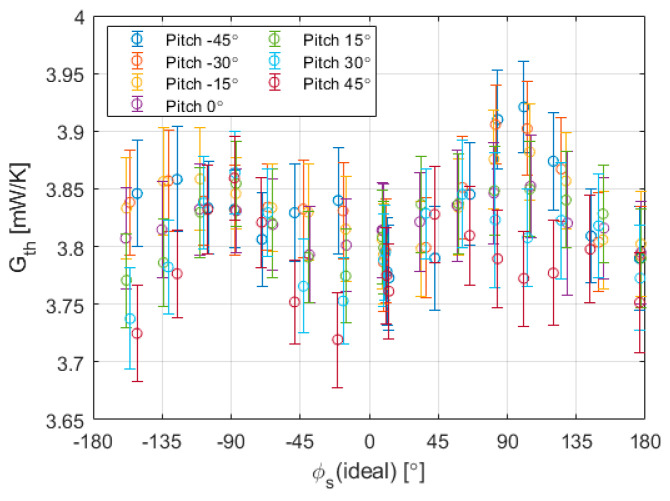
Estimation of the global convective heat transfer coefficient for the Aarhus experiment reported in Figure 11 and Figure 12. The experiment was carried out at constant wind velocity of 5.6 m/s, 49 °C of temperature overheat and 10 mbar of pressure.

Finally, the global convective heat transfer coefficient of the sphere can be evaluated all along the cited experiment. The estimation can be seen in Figure 18.

The inverse algorithm for the wind speed can be based on calibration or in the modeling explained in Section 2. For instance, considering an uncertainty of ±0.15 mW/K and the wind speed of the experiments reported in Figure 11 and Figure 12 (the slope in Figure 9 at 5.6 m/s is ~0.22 mW/K/(m/s)), this would result in an accuracy of 70 cm/s. On the other hand, the noise induced by the control electronics is smaller than ±0.05 mW/K (see Figure 18), and therefore a resolution below 30 cm/s can be stated for this case. It should be noted that these experiments have been performed with a system based on discrete component cards and using a first version of the inverse algorithm. Among the work in progress is the design and implementation of an ASIC, capable of controlling simultaneously two spheres, and the optimization of the algorithm. Therefore, it is expected that this will improve the resolution and accuracy of the sensor.

## 5. Experiment Set 2: Sensor Performance under Extreme Martian Conditions

Having verified the good performance of the spherical 3D sensor under typical Martian conditions, the next objective is to investigate its performance under extreme Martian winds, in which turbulent regimes may appear. To this end, both simulations and experiments of the sensor under wind speeds up to the Dust Devil scale and beyond have been performed.

Specifically, direct numerical simulations (DNS) of Navier–Stokes and energy equations for the sensor have been carried out for winds implying Reynolds number regimes up to Re = 1000. For higher wind regimes, up to Reynolds number Re = 10,000, Large Eddy Simulations (LES) have been used. The flow is solved in a domain of dimensions x = [−5.5D; 25.5D], r = [0; 10D] and φ = [0; 2π], with the sphere located at [0, 0, 0]. Meshes used for solving the flow have been constructed so as the boundary layer is well resolved, and the computational nodes are clustered in the wake of the sphere and close to the surface. Thus, the largest mesh used was 5.6 × 10^6^ grid-points. With this mesh, it was shown in [45] that it is possible to perform DNS at Re = 1000 and LES at Re = 10,000. The governing equations were solved by means of a low-dissipation finite element scheme, which preserves energy, momentum and angular momentum and discrete level. More details about the domain, meshes and numerical model and methodology used can be found in [45].

The simulations have been performed at MareNostrum IV Supercomputer, located in Barcelona. The main objective of these simulations is to predict what specific signatures, if any, should be observed in the power signals of the sectors. In the laminar regime shown in the previous section, the power signals are sinusoidal. However, if the Reynolds number regime is increased, changes in the shape of the signals may be expected.

To analyze the sensor response outside the laminar regime, an experimental campaign has been made for winds in the Reynolds numbers 1000 and 2000. For typical Mars conditions and taking into account the dimensions of the sensor, these values correspond approximately to wind velocities of 65 and 130 m/s, respectively. These experiments have been performed at the UPC hypobaric chamber and wind tunnel facility described in the next sub-section.

**Figure 19:**
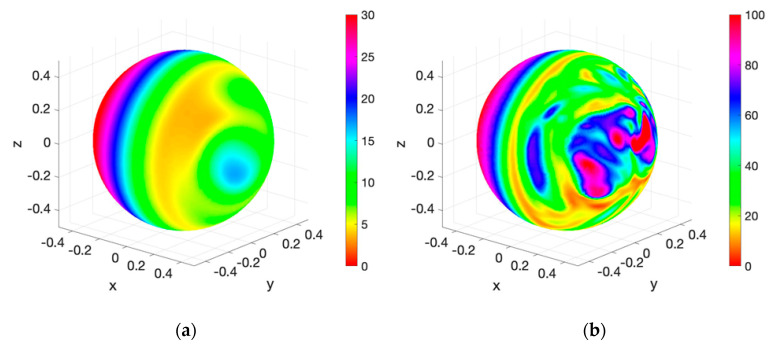
Instantaneous-local Nusselt number on the rear surface of the sphere obtained from direct numerical simulations (DNS) at Re = 1000 (**a**) and from LES at Re = 10,000 (**b**). The wind direction is parallel to the *x*-axis.

Figure 19 shows the simulation results, in terms of the Nusselt number at each point on the sensor surface (here considered as a continuous sphere), for the two aforementioned wind values: 65 m/s (Figure 19a, DNS) and 650 m/s (Figure 19b, LES). At these Reynolds numbers, the boundary layer is laminar up to separation from the sphere, and transition to turbulence takes place in the separated shear layers. For the larger Reynolds number, Re = 10,000, transition to turbulence occurs closer to the sphere surface thus affecting the heat transfer from the sphere at the rear zone. In the figure, the views shown correspond with the rear end of the sensor [45]. It is easily seen that turbulence effects are barely visible in Figure 19a, but clearly noticeable in Figure 19b. These effects can be seen as random peaks or spots in the instantaneous Nusselt number in Figure 19b as opposed to the smoother distribution observed at Re = 1000, in Figure 19a.

The results of simulations and sensor experiments for Reynolds numbers up to 1000, where the influence of turbulence and other non-ideality phenomena are just expected to appear, are presented and discussed in Section 5.2. Then, Section 5.3 does the same for higher Reynolds number values, up to the Dust Devil scale and beyond, where those phenomena are expected to become much more relevant.

### 5.1. Experimental Setup

**Figure 20:**
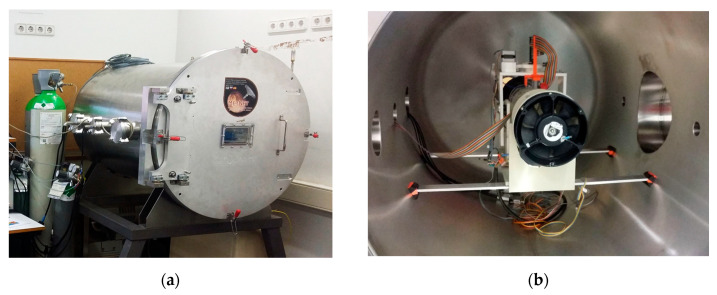
(**a**) High vacuum chamber and wind tunnel facility at the UPC; (**b**) Detail of the interior of the vacuum chamber. In the foreground is the fan that generates the wind inside the tube. The sensor is mounted, turned upside down, on the orange support, so the spherical head stays inside the tube. This support structure allows both changing the yaw and pitch angles of the sensor.

The UPC hypobaric chamber and wind tunnel facility consists of a 75 cm diameter and 120 cm length stainless steel cylinder, with a mini wind tunnel model WTM-1000 inside. A fan allows obtaining uniform flows within the 10 cm diameter section of the mini tunnel, which are controlled by the DC power provided. The combination of a rotary and a root vacuum pump, allows fast achieving of pressures as low as 0.1 mbar, well below those typical on Mars. Pictures with details of the vacuum chamber and the wind tunnel are shown in Figure 20.

There is no temperature control, therefore the system works at room temperature. Under these conditions, Mars-like wind flow at a given low temperature in CO_2_ atmosphere is emulated by reproduction of the same Reynolds number through adjustment of air pressure and air speed inside the chamber at room temperature, following the procedure described in [53]. Pressure and ambient temperature inside the chamber are continuously monitored during the experiments. Both the sensor interface and the PWM–based control strategy are the same as in the experiments presented in the previous section.

Let us remember that for the high regimes considered in these experiments, an unwanted effect that can decisively affect the behavior of the sensor is the turbulence caused by the grooves that separate the sensor sectors. To reduce this effect as much as possible, in the sensor used here, the grooves between the sectors have been filled with an insulation layer.

### 5.2. Towards the Dust Devil Scale: Reynolds Number Re ≤ 1000

**Figure 21:**
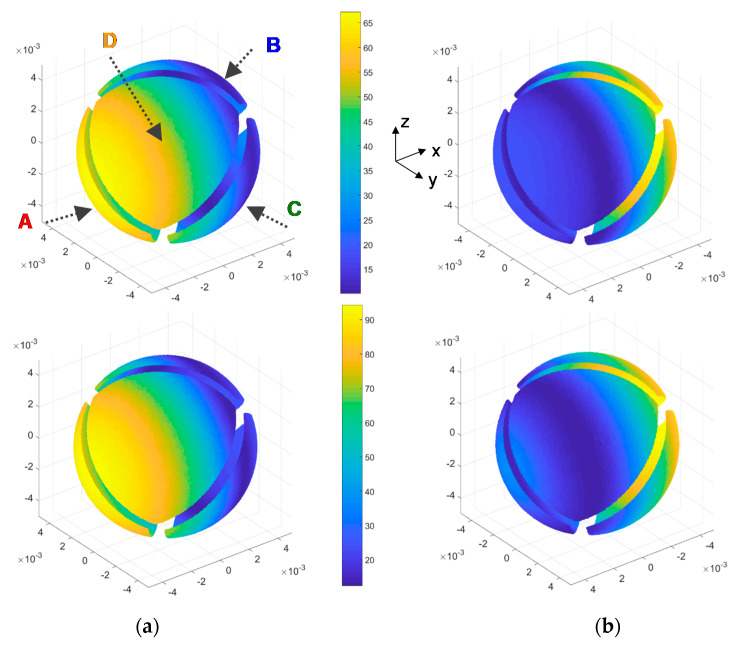
**Top**: Convective heat transfer coefficient (W/(m^2^K)) on the sector surfaces of the sphere, obtained from DNS at Re = 500; (**a**) is the front view, whereas (**b**) is the rear view. **Bottom**: same result obtained for Re = 1000. The wind direction is parallel to the *x*-axis. The disposition of the sectors A, B, C and D is the same the one previously shown in Figure 11, Figure 13, Figure 14 and Figure 15.

Figure 21 shows the DNS results of the convective heat transfer coefficient *G_th_* on the surface of the spherical sensor at both Re = 500 and Re = 1000, which correspond to wind speeds under typical Mars conditions of 32.4 and 65 m/s, respectively. Following the same strategy as in the simulations reported in Section 3.2 above and as it is shown in Figure 21, the four-sector structure of the sensor has been taken into account by eliminating the surfaces corresponding to the grooves which separate the sectors. The results are those expected for a quasi-laminar regime, i.e., the maximum to minimum values clearly follow the *x*-axis, but a certain stagnation area appears to be observed at the rear side of the sensor.

The objective now is to analyze how the average *G_th_* on a sector of the sphere changes as a function of the yaw angle. This will provide information on the expected behavior at each sector of the sphere when the sensor rotates. Following the same strategy as in previous simulations, this has been done through separate integration of the heat flux over the four sectors of the sensor.

**Figure 22:**
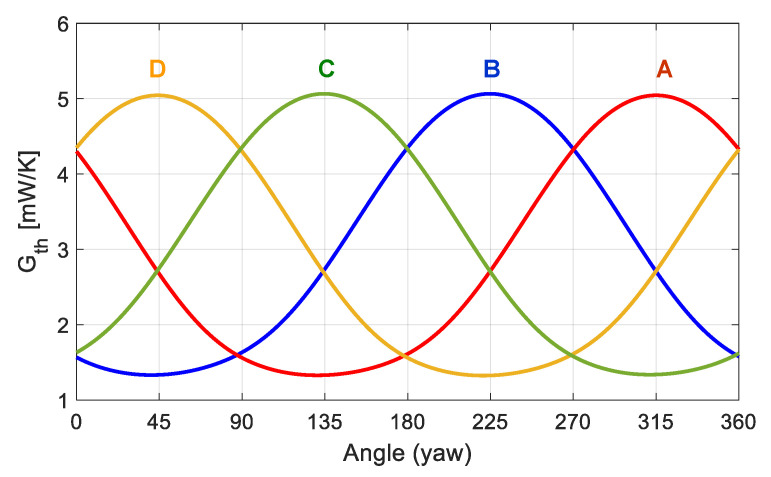
Evolution with the yaw angle of the average convective heat transfer coefficient *G_th_* of each sector of the sphere at Re = 1000. These values have been obtained by integration from DNS results. Sector names correspond to those defined in Figure 21. The disposition of the sectors A, B, C and D is the same the one shown previously.

An example of the results obtained is shown in Figure 22, where the average convective heat transfer coefficient *G_th_* of each sector is plotted as a function of the yaw angle of the incident wind, for Re = 1000. The shape of these curves strongly resembles the experimental ones reported in Figure 11 and the simulations of Figure 13, thus indicating that fast and efficient recovery of wind speed and wind direction is expected with this sensor even under these high wind regimes. Anyway, some widening of the heat transfer minima can be observed at the yaw angles that place the corresponding sector near the stagnation point in the wake of the sphere. As it will be shown later, this effect is even more pronounced for increasing Re numbers and it represents a signature of the expected behavior of the sensor.

**Figure 23:**
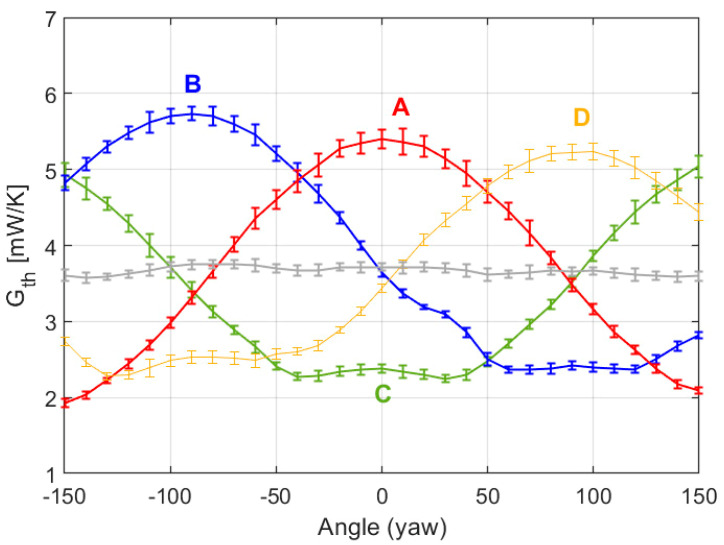
Experimental results showing the evolution with the yaw angle of the average convective heat transfer coefficient *G_th_* of each sector of the sphere (inferred from the average power delivered to each sector) at Re = 1000. The grey line is the average *G_th_* of the sectors. The 1σ error bars of the measurements are also shown. The disposition of the sectors A, B, C and D is the same the one shown previously.

On the other hand, Figure 23 presents the experimental curves of the convective heat transfer coefficient on each sector, obtained at Re = 1000 at the UPC facilities. The experiment parameters are the following: Equivalent Mars wind U = 65 m/s, Strouhal number St = f_VS_ D/U = 0.2 (where f_VS_ is the vortex shedding frequency, 1 kHz at Re = 1000), dry-air flow in the chamber of 6.5–7 m/s, pressure P = 250 mbar, ambient temperature *T_AIR_* = 22.9 °C, and target surface temperature *T_HOT_* = 35.6 °C. Due to limitations of the positioning system of the setup, it is only possible to scan yaw angles between ±157.5°, and the orientation of the sensor for yaw = 0 corresponds approximately to yaw = 315° in the experimental results of the previous section and the simulations.

Note that the experimental values and behaviors reported in Figure 23 are very close to those predicted by the simulations reported in Figure 22. In good agreement with the signature effect of the sensor predicted by the simulations, rear stagnation areas are observed. Note also that the average *G_th_* for the whole sensor (grey curve in Figure 23) is approximately constant throughout the experiment, which matches with the constant wind speed applied. Apart from the inevitable noise, it is observed that the heat transfer rate at the rearward point is higher in the experimental results than in the simulations. Most likely this is an effect associated with the PCB mast that supports the sensor, which produces a “shadow effect” from the incident wind, especially at the rear side of the sensor, increasing turbulence.

**Figure 24:**
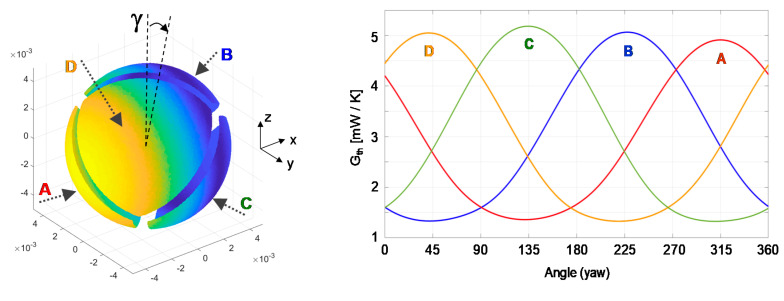
Evolution with the yaw angle of the average convective heat transfer coefficient of each sector of the sphere at Re = 1000 and a small tilt γ = 4.5° along the direction of the B-D groove. These values have been obtained by integration from DNS. The wind direction is parallel to the *x*-axis. The disposition of the sectors A, B, C and D is the same the one shown previously.

Another difference is that in the experimental results the vertical distance between maxima and minima seems slightly different in some sectors. This effect can be attributed to small misalignments of the sensor position, as in the example shown in Figure 24. This figure corresponds to the same case as in Figure 22, but assuming that the sensor has a small deviation γ = 4.5° in the direction of the gap that separates sectors B and D.

Summarizing, the results obtained allow us to affirm that, even under the hard wind conditions at Re = 1000, which may include a variety of undesired effects not present under laminar regimes, the spherical sensor will allow to recover the information about the speed and the direction of the incident wind, using an additional step of calibration to account for the particular shape of the curves. As an example, a calibration in [35] allowed the reduction of the shadowing effect of the ultrasonic sensor, producing similar distortions in the sensor signals, to within 1%.

### 5.3. Reaching and Surpassing the Dust Devil Scale: Reynolds Number Re > 1000

**Figure 25:**
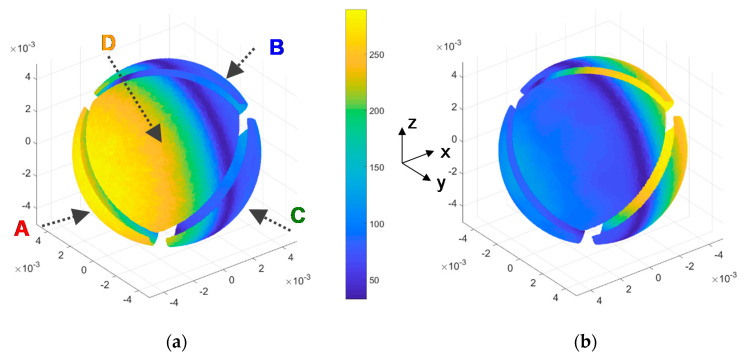
Front view (**a**) and rear view (**b**) snapshots of the convective heat transfer coefficient (W/(m^2^K)) on the sector surfaces of the sphere, obtained from LES at Re = 10,000. The wind direction is parallel to the *x*-axis. The disposition of the sectors A, B, C and D is the same the one shown previously.

For Reynolds number regimes beyond 1000, noticeable increases of the turbulence effects, which produce larger fluctuations in the local heat flux and other variables of the sensor are expected. Figure 25 shows the LES results of the local convective heat transfer coefficient on the surface of the sensor at Re = 10,000, an extreme condition that corresponds to a Mars-like wind speed of 650 m/s. Noticeable fluctuations due to turbulence are now clearly observed, especially when compared to the DNS results at Re = 500 and Re = 1000 of Figure 21.

**Figure 26:**
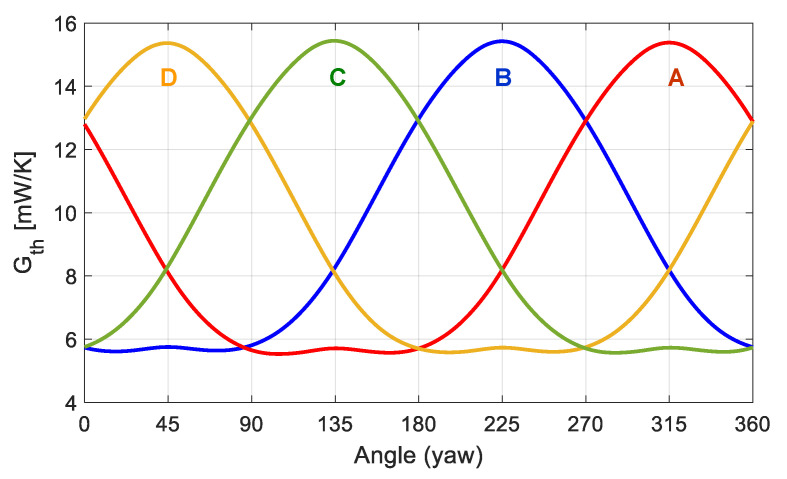
Evolution with the yaw angle of the average convective heat transfer coefficient *G_th_* of each sector of the sphere at Re = 10,000. These values have been obtained by integration from LES results. Sector names correspond to those defined in Figure 25. The disposition of the sectors A, B, C and D is the same the one shown previously.

Additionally, following the same approach as in the previous sub-section, Figure 26 shows the average convective heat transfer coefficient of each sector as a function of the yaw angle at Re = 10,000, as obtained from integration on each sector surface of the LES data. Again, the shapes of the curves strongly resemble the experimental ones of Figure 11, thus indicating that recovery of wind speed and direction is still possible with the sensor operating even under extreme wind regimes. The stagnation areas in the rear region are also observed, but with some differences: The stagnation area is widened and a local maximum between two minima is observed.

**Figure 27:**
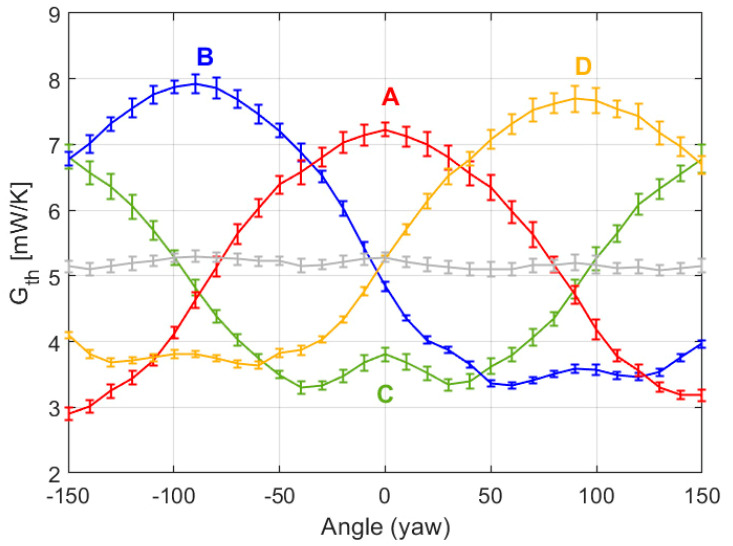
Experimental results showing the evolution with the yaw angle of the average convective heat transfer coefficient *G_th_* of each sector of the sphere (inferred from the average power delivered to each sector) at Re = 2000. The grey line is the average *G_th_* of the sectors. The 1σ error bars of the measurements are also shown. The disposition of the sectors A, B, C and D is the same the one shown previously.

On the other hand, Figure 27 reports the experimental convective heat transfer coefficient on each sector, obtained at Re = 2000 at the UPC facilities. The experiment parameters and conditions are the following: equivalent Mars wind U = 130 m/s, dry-air flow in the chamber of 6.5–7 m/s, pressure P = 500 mbar, ambient temperature *T_AIR_* = 23.9 °C, and target surface temperature *T_HOT_* = 34.4 °C.

The results shown in Figure 27 demonstrate that experimental results are in good agreement with LES: The stagnation and peak effect due to turbulence in the rear side of the sphere becomes more evident at Re = 2000. One of the major differences between the simulations and the actual sensor is the supporting structure (the two superimposed PCBs). The local maximum observed in the measured convection transfer coefficient when the sectors go into the wake of the sphere seems to indicate the presence of more turbulence than that predicted by the simulations. This “second maximum” effect is seen in the simulations only for the case Re = 10,000. A possible explanation is the presence of the supporting structure, and also the small rugosity once the grooves have been covered by the isolator.

These results point in the direction that successful 3D wind speed measurements can be made with the spherical sensor, even under these extreme regimes.

## 6. Conclusions

The performance of a miniature 3D spherical wind sensor designed for Mars atmosphere has been investigated. The sensor is a low power and low mass spherical structure divided in four sectors. By forcing the sectors to work at constant temperature, above that of the surrounding air, the 3D wind velocity components can be measured. The inference is made from the average powers that must be injected on the sectors to maintain the target temperature.

The results from an experimental campaign performed under typical Mars conditions at the Aarhus Wind Tunnel Simulator demonstrate that both wind speed and wind angle can be efficiently and reliably measured under Martian conditions (low pressure and low temperature). A very simple inverse algorithm has been presented. The accuracies obtained in the experiments of this campaign are 70 cm/s and 13°, with resolutions below 30 cm/s and 2°. These preliminary results, obtained with discrete component circuitry cards, are expected to improve using the ASIC and the new optimized versions of the algorithm that are currently under development.

The performance of the sensor under extreme Martian wind conditions, reaching and surpassing the Dust Devil scale has also been investigated, both through simulations and experiments. High-fidelity Navier–Stokes and energy-equations simulations have been carried out for Reynolds number regimes up to 1000, whereas Large Eddy Simulations have been used for higher winds, up to Reynolds number 10,000. The results of the simulations allow to predict the specific signatures that should be observed in the power signals of the sectors of the sensor under non-laminar, or turbulent, regimes. The predictions are further confirmed by the experimental results obtained for winds in the Reynolds number 1000 and 2000 regimes, which correspond to 65 and 130 m/s of wind speed under Mars conditions. These results demonstrate that wind speed and angle can be successfully recovered with this spherical sensor, even under such extreme regimes.

## Figures and Tables

**Table 1:** Physical dimensions and masses of the sensor components.

Component	Size	Mass
Sensor head	Ø 10 mm	3 g
2 × PCB	230 × 35 mm	12 g
PCB connector	55 × 25 × 8 mm	7 g
